# Functional imaging of hippocampal layers using VASO and BOLD on the Next Generation (NexGen) 7T Scanner

**DOI:** 10.1101/2025.08.29.673151

**Published:** 2025-09-04

**Authors:** Suvi Häkkinen, Alexander Beckett, Erica Walker, Laurentius (Renzo) Huber, David A Feinberg

**Affiliations:** 1 Helen Wills Neuroscience Institute, University of California, Berkeley, Berkeley, CA, United States; 2 Advanced MRI Technologies, Sebastopol, CA, United States; 3 NIH, Bethesda, USA

**Keywords:** fMRI, 7 Tesla, hippocampus, cortical depth analysis

## Abstract

Spatial accuracy and venous biases are a central concern in mesoscale fMRI, with subcortical brain regions facing additional challenges due to lower contrast-to-noise ratio (CNR), high physiological noise, and complicated vasculature. Here, we optimized CBV VASO on the NexGen 7T scanner for layer-specific investigations of the hippocampus. The presence of venous biases in VASO and BOLD (from the same acquisition) was then compared by using an established autobiographical memory task. While VASO and BOLD based activation patterns converged at macroscale, layer-specific differences emerged in the hippocampal subiculum, consistent with venous bias in the inner layers of the subiculum which can be explained by the unique two-sided venous drainage. Further, both VASO and BOLD showed sensitivity to short blocks (elaboration > construction), revealing an anterior-posterior distinction consistent with stronger involvement of the posterior hippocampus. Hippocampal cortical connectivity revealed brain circuitry between subcortical and cortical regions. Thus, hippocampal fMRI allows mapping layer function with high accuracy, made possible by sequence timing optimization on the high performance NexGen 7T scanner. The improved MR imaging has been developed to enable precision mapping of subcortical brain gray matter. By capturing changes of neural information flow within and across the microcircuitry of the hippocampus, it can provide deeper insights into a number of neuropsychological phenomena and the early changes occurring in Alzheimer’s disease (AD) and mild cognitive impairment (MCI).

## Introduction

A central pursuit in mesoscale fMRI is to model activation flow in the columns and depths of gray matter (Felleman & Van Essen, 1991). So far, most efforts have focused on neocortical circuits, yet incorporating subcortical areas and circuits would be highly relevant for neuroscientific and clinical applications. The changes in the early and presymptomatic stages of Alzheimer’s Disease (AD), for example, are deeply rooted in the subcortex, where pathology initially targets specific small structures (e.g. locus coeruleus and hippocampal subfield CA1; De Flores et al., 2015; Mather & Harley, 2016) and specific cortical laminae (Braak & Del Tredici, 2020), and spreads in patterns strongly linked with structural and functional connectivity (De Flores et al., 2022; Franzmeier et al., 2020; Grieco et al., 2023; Lace et al., 2009; Ottoy et al., 2024). As such, 7T MRI with its higher SNR can be used to study AD by evaluating changes in brain structure and function with higher resolution that allows better resolving small structures, accurate subfield segmentation of hippocampus, and detecting microcircuitry changes with layer fMRI. Imaging hippocampal activity is extremely challenging due to its location far from the receiver coils, high levels of physiological noise, and its intricate functional and structural organization.

Human hippocampus is a critical structure with a layered organization with gray matter in parts less than 1 mm thick, complex curved shape, and functional specialization along short (Alahmadi et al., 2023; Chang et al., 2021; Read et al., 2024; Seok & Cheong, 2020) and long axes (Angeli et al., 2025; Dalton et al., 2019; Tang et al., 2020; Zheng et al., 2021), and studies emerging also on laminar functional differentiation (Ahmadi et al., 2024; Maass et al., 2014; Pfaffenrot et al., 2025; Warrington et al., 2025). In addition to the requirement for sufficiently small voxel size and detection sensitivity, hippocampal layer-fMRI faces the challenge of intricate subfield- and layer-dependent vasculature: blood may drain to either inner or outer surface depending on hippocampal region and individual anatomy (Duvernoy et al., 2005). A bias which makes it difficult to reliably localize BOLD activity in neurovascular coupled activation is that blood oxygenation changes are also in the cortical draining veins, creating a confound in differentiating changes in layer activity (Duvernoy et al., 1981; Olman et al., 2007). Therefore, contrast mechanisms that are inherently less sensitive to the large draining veins (Feinberg et al., 2008; Lu et al., 2003; Oshio & Feinberg, 1991) would be very attractive for hippocampal layer-fMRI, but remain challenging in practice.

VAscular Space Occupancy (VASO) contrast is able to localize neuronal activity with high spatial specificity based on changes in cerebral blood volume (CBV), primarily the dilation of arterioles, capillaries, and intracortical arteries (Akbari et al., 2023; Hillman et al., 2007; Iadecola, 1993; Peppiatt et al., 2006). The VASO contrast and its lesser sensitivity to large draining veins has been validated using animal models and invasive imaging techniques (Huber et al., 2021), and commonly used in cortical layer-fMRI. The gain in specificity comes, however, at the cost of lower signal-to-noise ratio (SNR) and contrast-to-noise (CNR) of roughly half compared to BOLD at 7T due to larger venous deoxyhemoglobin dependent changes compared to microvascular blood volume changes (Beckett et al., 2020; Huber et al., 2014, 2015). Thus, reaching sufficient detection power with VASO in submillimeter hippocampal imaging may be challenging. Further, the pulse sequence and the lower statistical power of the contrast often necessitate the use of relatively long TRs and block designs, limiting the types of neuroscientific inquiries. Recent research however shows promise in applying VASO in high-noise subcortical (Burak et al., 2022; Khazar et al., 2025; Rane et al., 2016) and auditory (Faes et al., 2023) regions as well as in rapid short TR experiments (Dresbach et al., 2023). In this work, we will apply VASO to study hippocampal layers at the Next Generation (NexGen) 7T scanner (Feinberg et al., 2023). The NexGen 7T incorporates many high-performance components specifically adapted for mesoscale imaging at 7T, including high-channel array coils and a head-only asymmetric gradient coil (“Impulse”) with ten times higher performance than the body gradient coil available on standard 7T systems. This advanced hardware on NexGen 7T allows for better optimization for high-resolution imaging in hippocampus, through improved acceleration performance and SNR from the high-channel array, and reduced EPI readout time using the shorter echo spacing allowed by the head gradient coil. Thus, the scanner allows the opportunity for further VASO sequence optimization.

The recall of autobiographical memories rich in detail and context reliably activates hippocampus in fMRI (Addis et al., 2007; Leelaarporn et al., 2024; Pfaffenrot et al., 2025). In the present study, we will apply an established autobiographical memory paradigm to assist in the interpretation and validation of VASO contrast results. We assess activation based on VASO and BOLD contrasts, from the same acquisition, in terms of 1) sensitivity to detecting hippocampal activation during an autobiographical memory task, 2) ability to detect venous bias through comparison of VASO and BOLD from the same acquisition, and 3) the feasibility of detecting temporal dynamics during memory retrieval, requiring sensitivity to shorter blocks. In addition, we assess their ability to resolve functional connectivity between hippocampal and cortical areas. Together these results will inform on the sensitivity and specificity of VASO and BOLD to hippocampal activity, as well as on their capability in serving different experimental setups.

## Materials and Methods

### Subjects

Six participants (5 female; age 23–44 years) were scanned for the main analysis, and two additional in vivo scan sessions were used to identify the capabilities and challenges of hippocampal layer-fMRI as well as to implement and validate the imaging protocol. The study protocol was approved by the office for protection of human subjects, UC Berkeley IRB, and each participant gave written informed consent before MRI data acquisition.

### Data acquisition

Data were collected on the NexGen 7T scanner equipped with the high performance Impulse head-only gradient coil “Impulse gradient” with maximum amplitude and slew rate (Gmax 200mT/m, SR 900 T/m/s) and a 64-channel receive array coil (Feinberg et al., 2023). CBV weighted functional data were collected using a VASO sequence (Huber et al., 2014) with a skipped-CAIPI 3D EPI readout (Stirnberg & Stöcker, 2021). On the NexGen 7T scanner, the shorter echo spacings achievable using the Impulse gradient reduced signal dropout in the temporal regions, and importantly allowed readout of the 3D image slab within a single inversion recovery (IR) cycle of blood labeling. Therefore a segmented approach across multiple IR cycles was not required, which reduced the phase artifacts arising from such a segmented acquisition (Supplementary Figure S1). The scan parameters were: Matrix Size 206×206×30, field of view (FOV) 175×163, slice thickness 0.84 mm, TE 13.6 ms, In-plane segmentation 3, GRAPPA 1×3_z1_, Bandwidth 1516 Hz, Echo Spacing 0.72 ms, TI1/TI2 939 ms/2097 ms, TR_vol_ 1.158 s, effective TR (nulled/not-nulled) 3.2 s. The FOV was positioned sagittally, covering the right hippocampus and frontoparietal cortex expected to also be sensitive to the task paradigm. For anatomical reference, a whole brain MP2RAGE scan was also collected (voxel size 0.75 mm isotropic, matrix size 300×300×208, TE 2.82 ms, TR 6000 ms, Partial Fourier 7/8, GRAPPA 3, TI1/TI2 800 ms/2750 ms).

### Autobiographical memory task experiment

The task paradigm was adapted from previous studies on fine-grained hippocampal activation, which have demonstrated stronger activation across hippocampal subfields during *autobiographical memory* (AM) compared to *mental arithmetic* (MA) task (Leelaarporn et al., 2024; McCormick et al., 2015; Pfaffenrot et al., 2025). In the AM task, participants were presented with a cue-word (e.g., coffee) and were instructed to recall an event from their personal past that took place less than three years ago and lasted less than a day. The participants were told to press a button when they had selected a memory and then silently think about the event in detail until the word disappears, as if experiencing it again. Unique cue-words were taken from the Clark and Pavio extended norms (Clark & Paivio, 2004) based on high Thorndike-Lorge frequency, imageability and concreteness, as originally in (Addis et al., 2007). In MA, participants were presented with a simple mathematical problem (e.g., 15 + 12; additions, subtractions, multiplications, divisions) which they were to solve, then press a button, and start iteratively adding 3 to the result until the instruction disappears. AM and MA tasks were presented in alternating blocks separated by short fixation blocks. Data collection was TR-locked to the beginning of each task or fixation block, and task blocks had duration of 6 TR and rest blocks one TR, leading to 19.3 s task blocks separated by 3.2 s fixation blocks. Functional data was collected in three runs of 11.6 min (total 35 min). To assess the reliability of functional responses, one participant was measured twice on different days (total 70 min).

Participants briefly practiced the tasks right before the scan. Task compliance during scanning was measured by recording response button presses and verbally confirmed after the scan. Five participants indicated their task performance consistently throughout data acquisition (>97% and 88% of trials in the AM and MA task, respectively; Supplementary Table S1), but button presses were by mistake not recorded for one participant. Data from all six participants was included in main analyses comparing AM and MA blocks, but the data from the participant without recorded motor responses was excluded from the task stage comparison.

### Hippocampal subfield segmentation and layer extraction

The MP2RAGE was used to estimate hippocampal inner and outer surfaces and segmentation of subfields subiculum, cornu ammonis (CA) 1–4, and dentate gyrus (DG) using HippUnfold (version 1.4.1; DeKraker et al., 2022, 2023). The surfaces (with an approximate vertex spacing of 0.5 mm) were then transformed to VASO space to avoid resolution loss due to data interpolation: Registration of VASO and MP2RAGE was initialized by cortical rigid boundary-based registration (Greve & Fischl, 2009) and fine-tuned in hippocampal regions with ANTs’ symmetric normalization (SyN) algorithm (version 2.5.0; Avants et al., 2008). The affine and nonlinear transforms were applied to inner and outer hippocampal surfaces to bring them to native functional space using Connectome Workbench (wb_command version 1.5.0, Marcus et al., 2011). Finally, 20 equi-volume surfaces between inner and outer surface and 5 surfaces extending outside these limits were generated (*wb_command-surface-cortex-layer*).

### Cortical layer extraction

Brain surfaces were reconstructed from high spatial resolution (0.75 mm isotropic) MP2RAGE scans using the submillimeter recon-all pipeline from FreeSurfer (Dale et al., 1999; Zaretskaya et al., 2018). Analogous to hippocampal surfaces, the white and pial surfaces were warped to VASO space using ANTS and used to generate 25 equivolume surfaces representing different cortical depths, with inner and outer depth represented by the third surface inside white and pial surfaces.

### Functional activation analysis

Nulled and not-nulled series were motion-corrected to mean volume of the first run (AFNI 3dVolreg). Noise reduction with distribution corrected PCA (NORDIC PCA; version 4/22/2021; Moeller et al., 2021; Vizioli et al., 2021) was applied to nulled and not-nulled images separately, on magnitude only (Knudsen et al., 2025). VASO contrast data was generated using dynamic division (Huber et al., 2014), which involves temporal upsampling to better align the series of images with and without blood nulling, and was also applied to BOLD data. This provided VASO and BOLD time-series data acquired in the same acquisition, which we then analyzed separately using the same pipeline (similar to e.g. Huber et al., 2015, 2018; Oliveira et al., 2022; Pizzuti et al., 2023). Because BOLD and VASO signal changes are opposite in sign, the VASO signal was inverted for easier comparison.

Initial 2–5 upsampled volumes were disregarded based on automatic estimation (*NonSteadyStateDetector* implemented in Nipype, Gorgolewski et al., 2011). Activation was estimated in native voxel space using general linear models (GLM) as implemented in Nilearn (Abraham et al., 2014). Analyses used two designs. The first design matrix had two regressors of interest (AM, MA), and a contrast was defined to contrast the two tasks. The second, three task regressor design, further split AM condition into separate construction and elaboration stages based on the button press during the trial, and a linear contrast compared these two memory task stages. For the second design, only trials with button press were modeled, and the one participant (S3) without recorded button presses was not analyzed. The average time of construction and elaboration stages were 3.2 and 16.2 s, respectively (see also Supplementary Table S1). Both models used a high-pass filter of 0.011 Hz. To confirm that results are not explained by physiological noise, we repeated analyses including the six head motion parameters, outliers (> 0.5 mm mean framewise displacement; Power et al., 2012), and physiological correlates from the aCompCor (Behzadi et al., 2007) variant described in (Pfaffenrot et al., 2025). This variant includes signals from a white-matter ROI (a 4 voxel cube manually placed in adjacent white matter; components chosen to explain 50% variance) and a high residual ROI (> 3 SD of all residuals from the GLM fit; 5 components) putatively sensitive to respiratory and cardiac cycles. The aCompCor regressors were orthogonalized with respect to motion regressors.

To assess layer-specificity of VASO and BOLD activation, statistics were sampled from volume to hippocampal surfaces (*wb_command-volume-to-surface-mapping*) using trilinear interpolation. To visualize the hippocampal the peaks and topographical patterns along both short and long axes of the hippocampus, activation was also plotted on unfolded hippocampal surfaces. The unfolded activation maps were also collapsed across subjects with a 2 mm Gaussian smoothing kernel sigma (*wb_command-metric-smoothing*) to assess group mean activation. Individual hippocampal surfaces were registered using a surface-based approach using HippoMaps (0.1.0, DeKraker et al., 2024). The software was also used to plot the unfolded canonical maps at middle, inner and outer layer (third surface hippocampal side of the boundary surface). Finally, to assess depth-dependent activation profiles, the vertexwise statistics were averaged per depth and subfield (subiculum, CA1, CA2, CA3). In this comparison of VASO and BOLD, we only included the middle surface of CA4 and dentate gyrus (DG) since the neuronal laminar organization of these subfields is less clear.

### Functional connectivity analysis

Connectivity analysis assessed connectivity patterns between areas of the hippocampus and part of the cortex within FOV. Data was preprocessed by regressing out task effects (blocks) and nuisance covariates (aCompCor, six motion parameters, motion outliers of > 0.5 mm FD), and high-pass filtering (0.01 Hz). Data was then sampled to hippocampal inner or outer depths (third surface inside white and pial surfaces). Vertices were then grouped based on subfield and belonging to the anterior or posterior hippocampus (35% and 65% of hippocampal length, respectively; Hackert et al., 2002), and time series defined by vertexwise mean. Background connectivity was then measured as Pearson correlation between seed time-series and brain voxels, and sampled to inner or outer cortical surfaces. To assess group-level patterns, surfaces were normalized using spherical surface registration (Fischl et al., 1999) and value overlays smoothed 3 mm FWHM on surface to account for individual variability (*mri_surf2surf*). Group-level patterns were characterized by one and two sample t-tests calculated using PALM (https://github.com/andersonwinkler/PALM; Winkler et al., 2014).

Functional connectivity based on VASO and BOLD were expected to show similarity in the spatial patterns of task-evoked activation, as they are strongly related to each other and functional boundaries (Gordon et al., 2016). Further, based on previous literature, we expected relative differences to emerge in the network connectivity of anterior and posterior hippocampus. As the subiculum is a major output region of the hippocampus with neocortical circuits arising primarily from the pyramidal cell layer (Aggleton & Christiansen, 2015; Lavenex & Amaral, 2000; Roy et al., 2017), we sampled the subiculum middle layer for this network assessment. Finally, to assess potential venous bias confounds, connectivity of inner and outer layers were compared in the subiculum (outer to inner venous bias) and CA3 (weak bias).

## Results

### VASO and BOLD activation to memory task

**Figure 1:**
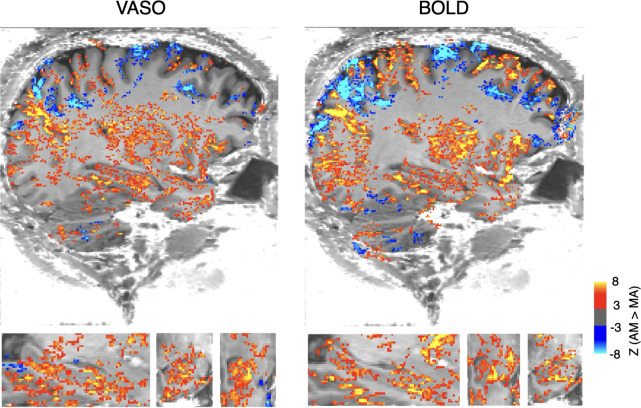
Consistent macroscale activation for VASO and BOLD during memory task (AM > MA). This analysis was based on two sessions (70 minutes) of data. For this macroscale visualization only, VASO and BOLD were denoised with NORDIC factor error levels of 1.5 and 1.0, respectively, to highlight the noisier VASO pattern. Both were thresholded at Z > 3 and clusters of 100 voxels (AFNI *3dClusterize*).

To assess the capability of VASO and BOLD in detecting hippocampal activation, we measured activation during an established autobiographical memory task paradigm. Contrasting autobiographical memory and mental arithmetic conditions revealed that, at macroscale, VASO and BOLD patterns converged in the cortex as well as hippocampus (Figure 1; for cortical group mean patterns see Figure 4a). As expected, BOLD had higher detection sensitivity as measured by individual level z-scores.

**Figure 2:**
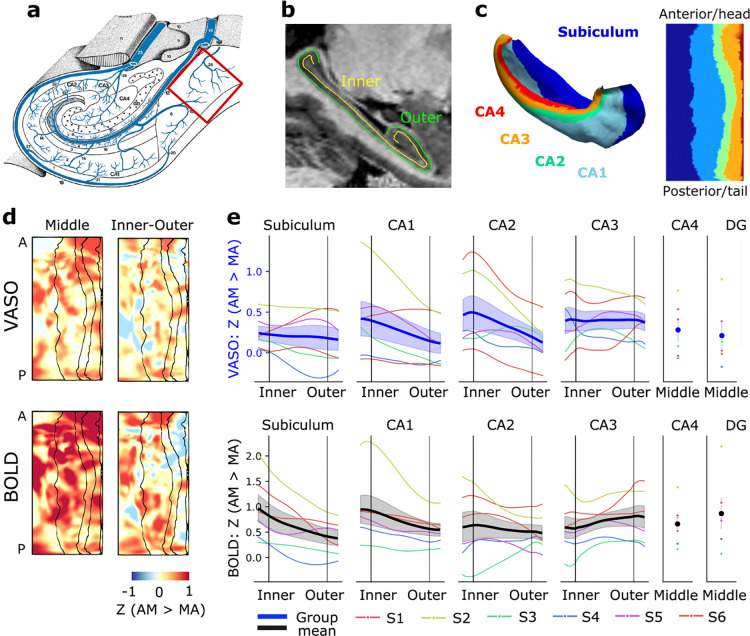
Layer-specific memory task activation (AM > MA) based on VASO and BOLD from the same acquisition. (**a**) Schematic of hippocampal venous drainage patterns (modified from Duvernoy et al., 2005), with the inner layers’ venous bias in subiculum highlighted. (**b**) Hippocampal inner and outer layers overlaid on sagittal MP2RAGE. (**c**) Hippocampal subfield segmentation on folded and unfolded hippocampal surfaces. (**d**) Group average activation patterns visualized on unfolded surfaces. Task activation sampled at middle layer shows putative anterior emphasis, and inner-outer difference maps show relatively stronger subiculum inner layer activation in BOLD compared to VASO. (**e**) ROI analysis of memory task activation per hippocampal subfield. Note the stronger activation in the inner layers of subiculum in the BOLD but not VASO results, consistent with a venous bias. In CA1, stronger inner layer activation is suggested by both contrast mechanisms. Thick line represents group mean and standard error.

For finer-grained analysis, hippocampus was segmented into layer and subfields, allowing focused analysis of the subiculum expected to have a clear venous bias (**Figure a–c**). Task activation patterns between VASO and BOLD converged at the level of hippocampal surface patterns (Figure 2d; for individual maps see Supplementary Figure S2). Activation was clearly stronger during autobiographical memory compared to math across hippocampal subfields, with several participants showing a putative anterior emphasis. Analysis of layer profiles (i.e., relative strength of response at different depths of gray matter) revealed subtle differences between VASO and BOLD (**e**). In line with the known venous drainage pattern in the subiculum to the inner surface (Duvernoy et al., 2005), the activation profile shapes revealed stronger BOLD activation during the memory task in the inner layers. The VASO–BOLD difference was seen quite systematically also at individual participant level, with an absent or reversed effect in VASO. The true venous bias in other subfields is less clear, though relatively stronger bias towards inner layers in CA1 and outer layers in CA2 and CA3 are often assumed. In our results, in CA1, both VASO and BOLD show stronger activation in the inner layers. In CA2 and CA3, BOLD compared to VASO showed relatively stronger outer compared to inner layer activation, which would be consistent with misplaced activation from inner to outer layers due to bias towards outer layers.

The relationships of VASO and BOLD in subiculum and CA1 were quite robust to preprocessing choices related to physiological noise regressors (Supplementary Figure S3) and NORDIC denoising (Supplementary Figure S4). Specifically, NORDIC denoising greatly facilitated the detection of VASO patterns in our hippocampal VASO dataset by increasing z-scores without fundamentally changing layer profile shape. Some effect sizes (namely beta estimates in the inner layers of CA1; Supplementary Figure S4) were however reduced, which may result from signal components not being separable from the thermal noise in high noise datasets (Faes et al., 2024; Kay, 2022; Olesen et al., 2023).

To test the reliability of data and VASO–BOLD distinctions in 35 min acquisitions, we collected two sessions on one representative participant. The key results of VASO–BOLD difference in the inner layers of subiculum and similarity in CA1 are visible in individual sessions as well as in a combined analysis (Supplementary Figure S5).

### VASO and BOLD activation to memory task stages

**Figure 3:**
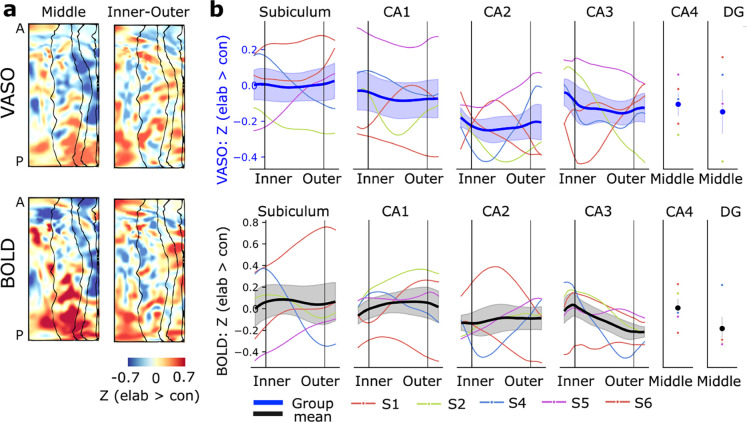
Layer-specific activation differences between the stages of memory task (elaboration > construction) based on VASO and BOLD from the same acquisition. (**a**) Group average activation patterns visualized on unfolded surfaces. Both VASO and BOLD showed stronger activation in the inner layers of the posterior hippocampus, particularly in the inner layers, during the stage with emphasis on memory retrieval. (**b**) ROI analysis of activation differences to memory stages per hippocampal subfield, with less apparent patterns. Thick line represents group mean and standard error.

Autobiographical memory recall exhibits a temporal dynamic between the initial construction and subsequent elaboration, with partially distinct neuronal correlates. These task stages could be compared by splitting the AM condition based on the button press in each trial, providing an additional test case for comparing VASO and BOLD activation; one involving faster task dynamics (reconstruction duration similar to our TR of 3.2 s; column “AM response time” in Supplementary Table S1). In our analysis on hippocampal surfaces, the comparison of reconstruction and elaboration showed an anterior-posterior distinction (Figure 3a; for maps of individual participants see Supplementary Figure S6), with the elaboration stage associated with relatively stronger activation in the posterior hippocampus and its inner layers. Layer profiles (averaged per subfield) did not reveal additional consistent differences across participants (**b**), probably due to the heterogeneity along the long axis.

### Hippocampal cortical connectivity

**Figure 4:**
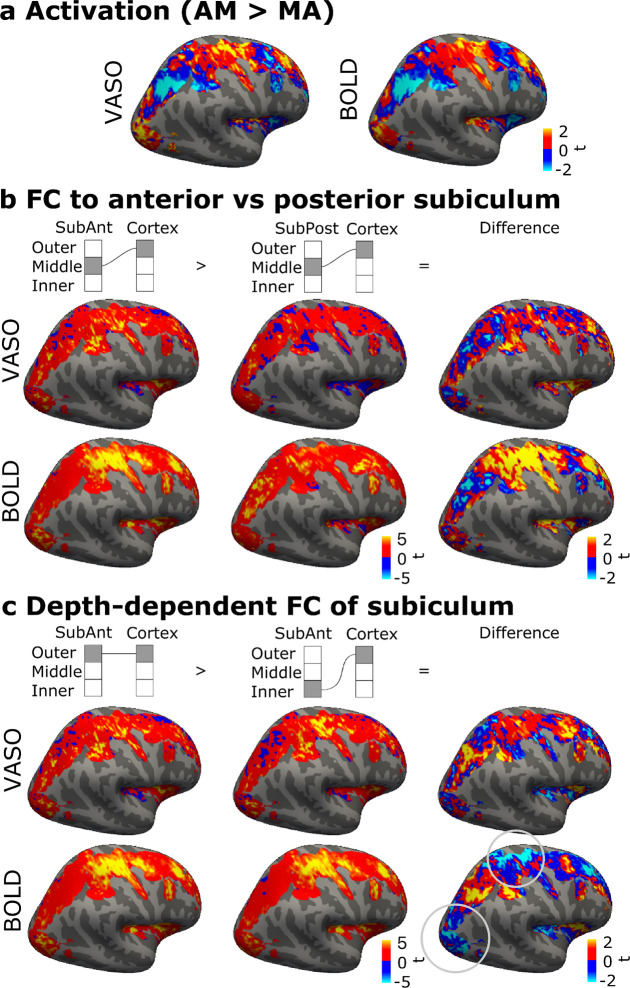
Cortical activation and hippocampal functional connectivity patterns based on VASO and BOLD, in the group of six subjects. (**a**) Converging activation patterns (AM > MA) indicated functionally distinct areas. (**b**) Functional connectivity (FC) patterns associated with anterior and posterior subiculum, a major output region of the hippocampus, showed relative anterior–posterior differences. (**c**) Connectivity seeded by inner and outer depths of anterior subiculum, a region with a known outer-to-inner venous bias. Depth-dependent differences based on VASO and BOLD show differences (circled). In (**a–c**), statistics are shown only for the right hemisphere vertices imaged in all six participants.

Functional connectivity patterns were expected to show some resemblance to the task-evoked activation patterns (Figure 4a). The cortical activation patterns were spatially fine-grained. At group level, activation was stronger during AM than MA task in prefrontal, visual and motor cortices (red). MA, in turn, showed stronger VASO and BOLD activation in for example inferior parietal regions (blue).

Comparison of the relative differences in the connectivity of anterior and posterior hippocampus (Figure 4b) showed that posterior subiculum more strongly connects to areas also activated for the AM than MA task. The spatial pattern was clearer in BOLD than VASO, likely explained by the lower SNR in VASO.

In the analysis of depth-dependent connectivity, both VASO and BOLD showed for example that connectivity to most cortical areas more activated by math (MA > AM) was stronger from the outer than inner layers of the subiculum (Figure 4c; red). Connectivity patterns were also visually quite similar, showing for example a clearly different depth-dependent connectivity pattern of CA3 compared to the other subfields (Supplementary Figure S7). The depth-dependent connectivity patterns obtained with VASO and BOLD however also showed differences. Depth-dependent patterns of the subiculum, with a known strong venous bias, showed pronounced VASO–BOLD differences. Notably, these differences occurred in areas showing stronger activation during memory task (blue in **a**), and all in the direction of BOLD suggesting stronger subiculum inner than outer layer connectivity. Thus, the difference might be driven by the venous signal reflected in BOLD based connectivity, misplacing signal fluctuations from outer to inner layers of subiculum leading to overestimation of inner-layer activity.

## Discussion

This study demonstrates that CBV VASO is an effective tool for investigating hippocampal circuitry, in the context of an established autobiographical memory task. Concurrently acquired VASO and BOLD differed in the suggested layer-resolution localization of activation and connectivity, consistent with a dominant venous signal in BOLD obscuring depth-dependent distinctions in activity. These findings characterize VASO as a valuable method to improve localization accuracy for hippocampal circuitry mapping in neuroscientific and clinical applications.

### VASO and BOLD differences in activation are consistent with venous bias in hippocampus

VASO and BOLD results converged well at the macroanatomical level, indicating activation in the hippocampus during autobiographical retrieval (AM > MA) (Figures 1), stronger activation (AM > MA) in the anterior than posterior hippocampus (Figure 2c; Supplementary Figure S2), and during elaboration compared to construction stage of the memory task (Figure 3a). As expected, however, VASO and BOLD results differed at layer resolution.

The dual contrast fMRI approach used here with VASO and BOLD extracted from the same acquisition allowed us to identify which BOLD layer profiles can be interpreted with respect to neuronal laminar circuitry and which profiles need to be considered as vascular biases. In particular, we planned to use subiculum as a test case, as previous literature is clear that the blood in subiculum is drained through veins along the inner surface (Duvernoy et al., 2005), with respective expected artifactual fMRI signal misplacement (Haast et al., 2024; Pfaffenrot et al., 2025). The activation results in the subiculum clearly show a VASO–BOLD mismatch with more activation in the inner layers using BOLD than VASO, implying less bias in VASO (Figure 2d). Thus, a BOLD based analysis might lead to the interpretation that input-receiving outer layers of the subiculum are less active, and the inner output layers more active, than they truly are.

Contrasting with the clearcut difference between VASO and BOLD results in the subiculum, results were quite different in CA1, which also has a prominent (outer to inner) vascular bias. In CA1, both VASO and BOLD contrasts indicated stronger activation in inner than outer layers (AM > MA; Figure 2d), suggesting that there is a true task-related activation difference between depths of CA1. This finding aligns with the previous layer-BOLD study (Pfaffenrot et al., 2025), which compared responses to the autobiographical task and hypercapnic challenge, and found them both associated with greater CA1 inner layer activation but with distinct laminar response shapes. They concluded that while venous bias is not the sole driver of this inner layer CA1 response, results in the area should be interpreted with caution. The observed depth-specific pattern is also in line with greater input to CA1 from the trisynaptic pathway (DG → CA3 → CA1), compared to the perforant path (entorhinal cortex → CA1), which terminate in inner and outer layers of CA1, respectively (Amaral & Witter, 1989). Specifically the trisynaptic pathway has been widely implicated in pattern completion such as retrieval of specific episodic memories (Rolls, 2013). In cases such as CA1 during autobiographical memory recall where neuronal and vascular responses are very similar, VASO contrast may provide a well-principled way to disentangle their relative contributions.

Our results do not indicate strong differences between VASO and BOLD in subfields CA2 and CA3, though several subjects showed relatively stronger activation in outer compared to inner layers (Figure 2d). The small outward bias in CA3 and lack of considerable bias in CA2 is in line with Pfaffenrot et al. (2025). We however cannot rule out large individual variability or weak task activation making the distinctions between VASO and BOLD difficult to detect in the current experiment. Weaker task activation could relate to the autobiographical memory task not strongly engaging these subfields (Leelaarporn et al., 2024), or the lower activity sparsity (proportion of active neurons in a population) in these subfields (Lee et al., 2025; Watson et al., 2025).

Results based on the two contrasts also agree on the specialization along the hippocampal long axis, with both suggesting an anterior emphasis for autobiographical memory retrieval (Figure 2c). The comparison of elaboration and construction stages revealed further nuance: activation during elaboration was relatively stronger in the posterior than anterior hippocampus, and in the posterior hippocampus stronger in inner than outer layers (Figure 3a). The posterior hippocampal emphasis to elaboration is consistent with previous studies contrasting these task stages (Audrain & McAndrews, 2022; Pfaffenrot et al., 2025) as well as with the theory that posterior hippocampus supports the retrieval of fine-grained details and anterior hippocampus coarser, gist-like memory features (Audrain & McAndrews, 2022; Brunec et al., 2018; Poppenk et al., 2013; Sekeres et al., 2018). The relative difference between the inner and outer layers of several posterior subfields is also in line with findings of the previous layer-BOLD study (Fig. S10K in Pfaffenrot et al., 2025). A possible circuitry level explanation might be continuous engagement of hippocampal pattern completion as more details are matched to the episodic event during elaboration: e.g., input from dentate gyrus to the inner layers of CA3 for pattern completion and from CA3 to the inner layers of CA1.

### Neocortical activity and hippocampal connectivity

Hippocampus and neocortex are known to function in concert during memory retrieval. As our FOV captured some of the relevant neocortical areas, we were able to assess their interplay with the hippocampus. Based on visual comparison to known large-scale cortical network patterns, autobiographical memory was associated with stronger activation in areas likely overlapping with putative default mode or parietal memory networks (Figure 4a, red), strongly associated with autobiographical memory (Buckner et al., 2008; DiNicola et al., 2020; Kwon et al., 2025), as well as in visual and motor cortices, which may reflect sensory reactivation (Gilmore et al., 2021; Hofstetter et al., 2012). The mental arithmetic task, in turn, showed stronger VASO and BOLD activation (blue) in frontoparietal network areas such as intraparietal sulcus (Dehaene et al., 2004; Matejko & Ansari, 2021).

The anterior emphasis in hippocampus for autobiographical memory retrieval (Figure 2c) is consistent with previous literature suggesting stronger anterior than posterior hippocampal connectivity to the default mode network (Angeli et al., 2025; Borne et al., 2023; Tang et al., 2020; Vos De Wael et al., 2018; Zeidman & Maguire, 2016; Zheng et al., 2021). Posterior hippocampus, in turn, has been reported to have relatively stronger connectivity to parietal memory, visual and sensorimotor networks, though cortical connectivity may also vary by hippocampal subfield (Ezama et al., 2021; Leelaarporn et al., 2024; Warrington et al., 2025). In this context, the finding that elaboration compared to construction was associated with stronger activation in the posterior hippocampus (Figure 3a) might relate to pronounced involvement of the parietal memory network, sensitive to familiarity and goal-oriented cognition. Further study of these hippocampal neocortical connections might help better characterize the memory-related networks.

To summarize, cortical (macroscale) task activation based on VASO and BOLD converged (AM > MA; Figures 1 and 4a). Spatially similar patterns also emerged in comparisons of functional connectivity of anterior and posterior hippocampus (Figure 4b, Supplementary Figure S7) and inner and outer layers of the subiculum (Figure 4c). This sensitivity to different aspects of functional organization suggests that both contrasts can be used for mesoscale functional connectivity mapping. However, depth profiles of functional connectivity based on VASO and BOLD differed in the subiculum (Figure 4c) such that BOLD showed pronounced cortical connectivity associated with the inner layers. Given that this difference is in the direction of potential BOLD signal displacement in the subiculum, it serves as a cautionary example on venous confounds in BOLD also in functional connectivity.

### Impact of NexGen 7T scanner on mesoscale functional imaging

These methodological advances have exciting implications for noninvasive, in-vivo mapping of the microcircuits to and from the hippocampus in humans. Optimization of VASO imaging in the hippocampus required us to leverage the advantages of the high signal at ultra-high field and the unique hardware of the NexGen 7T scanner (Feinberg et al., 2023) to achieve high-resolution CBV-weighted imaging with sufficiently short TR to resolve the different substages of autobiographical memory retrieval, and obtain sufficient SNR to overcome the reduced sensitivity of VASO when compared to BOLD. On standard 7T scanners, one or more of these parameters would need to be compromised for VASO imaging in this subcortical brain region. This novel hardware is being further optimized for mesoscale imaging and will become more available at other research centers. Outstanding challenges include expanding spatial coverage without sacrificing resolution or sensitivity, which would allow for functional connectivity analyses to infer information flow between close (e.g. entorhinal cortex, Koster et al., 2018; Zhang et al., 2023, or parahippocampus, Warrington et al., 2025) and distant cortical regions involved in primary sensory and executive control networks.

### Impact of vein-free layer-fMRI in the hippocampus in future applications

Vein-free laminar imaging of the hippocampus opens the door to investigating computational mechanisms behind any number of neuropsychological phenomena:

- Imaging layers of the hippocampus are critical for investigating neural correlates of episodic memory to understand how it orthogonalizes representations that minimize interference between experiences and also integrating information across episodes (Koster et al., 2018).
- While it is known that the hippocampus plays a crucial role in spatial navigation, the underlying circuit-level mechanisms are poorly understood (Ahmadi et al., 2024).
- Specific subtypes of epilepsy, such as Type III of focal cortical dysplasia (FCD) can be caused by cortical dyslamination associated with hippocampal sclerosis (Aitken et al., 2025).
- The layers and subfields of the hippocampus are differentially affected in dementia, particularly in Alzheimer’s disease. Specifically CA1 region and entorhinal cortex layer II are critical early sites of damage and are linked to memory impairments (Gómez-Isla et al., 1996; Kerchner et al., 2012). Imaging the layered structure of the hippocampus can be a useful research tool to capture the development and propagation of this pathology in the living human brain.
- Mood and stress disorders show involvement of specific subfields and layers such as a retraction of CA3 dendrites, decreased neurogenesis and structural connectivity of dentate gyrus, and decreased dentate/CA3 BOLD activation (Leal & Yassa, 2018; Rutland et al., 2019).
- Post-mortem studies on schizophrenia consistently report alterations in the neuronal morphology and synaptic density in the hippocampus (Harrison, 2004). Emerging theories postulate layer-specific mechanisms, such as hallucinations explained by dysfunction in integrating predictions with sensory evidence (Haarsma & Kok, 2025).

In general, the vascular organization of hippocampus and other subcortical regions is less charted, but drainage patterns are less straightforward compared to the neocortex and there is variability between participants (Duvernoy et al., 2005; Haast et al., 2024; Spallazzi et al., 2019). The venous confound may introduce noise and mislead conclusions on the directionality of information flow based on BOLD data (especially in areas where the direction of the bias is unknown or varies between participants), whereas VASO may provide an additional insight to neural activity. The ability to do vein-free imaging of the hippocampus can also be used to validate when BOLD localization of neural activity can be judged to be accurate (e.g., memory related activation in the inner layers of CA1, despite a likely bias in the same direction).

Looking beyond the hippocampus, the tools developed here provide a starting point for mapping layer-specific connections within lower brain areas of cortex and between neocortex and sub-cortex, in the context of cognitive neuroscience, clinical neuroscience, and neurology. Until now, more than 95% of all >300 published human layer-fMRI studies could solely focus on brain areas in the upper half of the brain (source: layerfmri.com/papers), limiting the potential to capture neural information flow across laminar microcircuits throughout the entire human brain. The methodology developed here helps the field overcome these limitations and finally address questions of many influential theories of brain function that posit laminar signals with origins and destinations in distinct cortical layers and lower brain areas. Several exemplary theories such as Predictive Coding, Thalamocortical Loop Models, Limbic–Cortical Integration Models for Emotion and Decision-Making may now be directly tested in humans. We expect that the ever-advancing tools and improvements of high-resolution fMRI will ultimately transform our understanding of cognition in the awake, behaving human brain.

CBV based fMRI has been shown to provide high specificity in imaging cortical and hippocampal layer activity, useful for identifying neurocircuitry changes in neurological disorders in humans. The presented techniques will enable non-invasive imaging of neuronal circuits in the human brain and studies of connectivity and microcircuitry changes in AD and related dementias.

## Conclusion

VASO sequence optimized for the NexGen 7T scanner was sensitive to hippocampal activation during autobiographical memory. The depth-dependent activation differences based on VASO and BOLD were consistent with known hippocampal vasculature and highlight lesser venous bias in VASO. While several task activation effects were observed quite similarly using both contrasts, others were obscured by the venous biases in BOLD, producing clear mislocation (subiculum) and possible artifactual amplification (CA1) of task effects of interest. The differences consistent with venous confounds in BOLD extended to hippocampal depth-dependent functional connectivity to the neocortex. VASO is therefore a strong alternative for studies on circuit mechanisms where cortical depth-specificity is critical.

## Supplementary Material

Supplement 1
