# Supplementary material for: Functional imaging of hippocampal layers using VASO and BOLD on the Next Generation (NexGen) 7T Scanner
Source: bioRxiv. 2025 Sep 4:2025.08.29.673151. Preprint. [Version 1] doi: 10.1101/2025.08.29.673151 (PMC12424771; doi:10.1101/2025.08.29.673151)
Supplement: Supplement 1 [file media-1.pdf]

Supplementary materials for: Functional imaging of hippocampal layers using VASO and BOLD on the Next Generation (NexGen) 7T Scanner

Suvi Häkkinen[1*], Alexander Beckett[1,2], Erica Walker[1,2], Laurentius (Renzo) Huber[3] and David A Feinberg[1,2]

[1]Helen Wills Neuroscience Institute, University of California, Berkeley, Berkeley, CA, United States,
[2]Advanced MRI Technologies, Sebastopol, CA, United States,
[3]NIH, Bethesda, USA

Table S1. Task performance during fMRI acquisition.

Participant	AM responses	AM response time (s)	MA responses	MA response time (s)
S1	100%	1.61	100%	1.93
S2	97.77%	5.93	88.89%	11.39
S3	NA	NA	NA	NA
S4	100%	2.36	97.77%	4.38
S5	100%	3.73	100%	3.36
S6	100%	2.49	100%	6.41

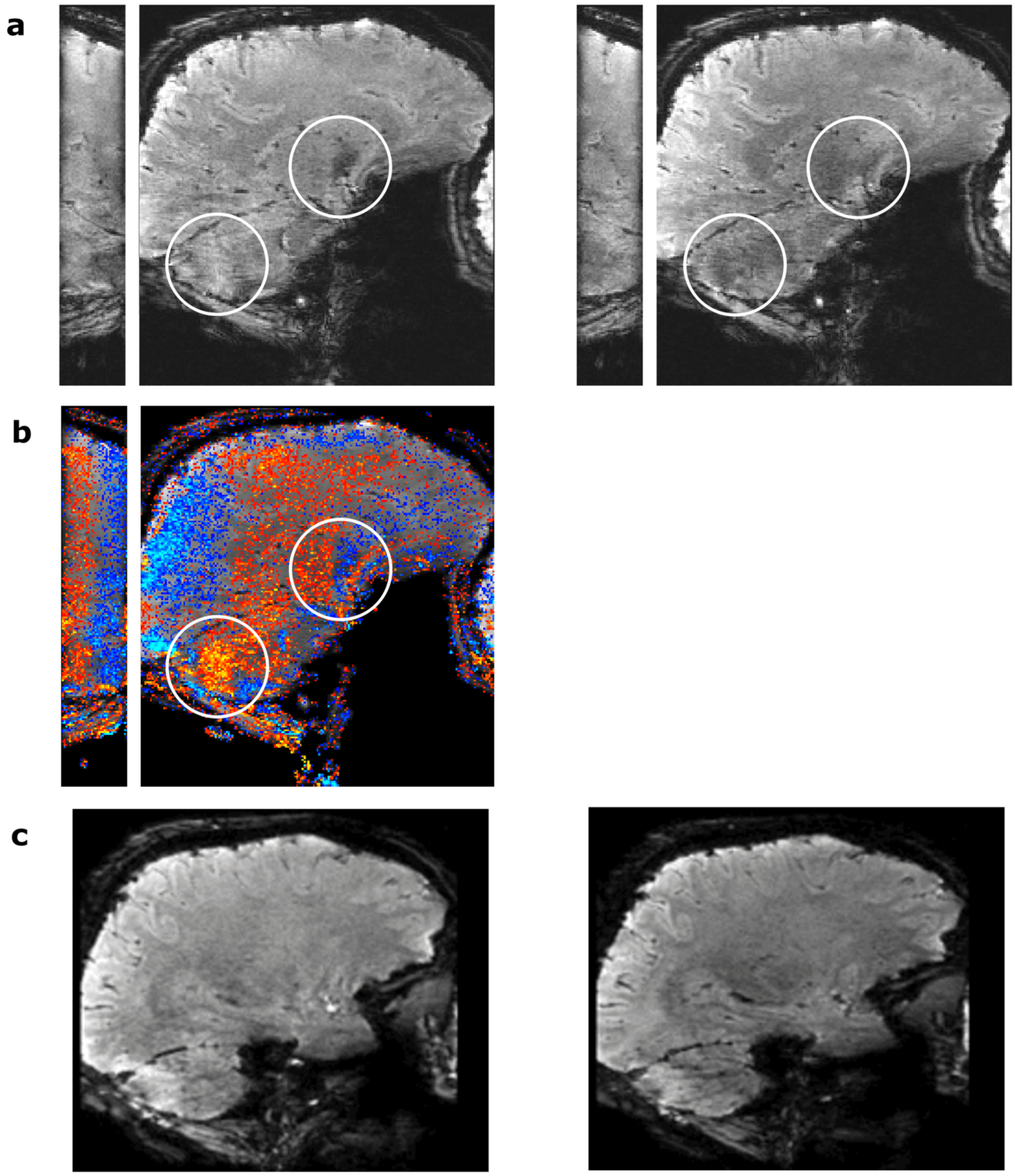

Figure S1 Examples from initial testing stage. (**a**) Two consecutive Nulled volumes acquired using a segmented acquisition scheme, showing a phase artifact from combining two different inversion recoveries to an image in the left-right direction. (**b**) The same phase artifact leading to artefactual activation differences in task activation z-scores. (**c**) The shorter echo spacings available on NexGen 7T scanner (0.72ms (1516 Hz) vs 0.97ms (1106 Hz) achievable on a typical 7T Terra) also helped reduce signal dropout in the medial temporal regions, as shown in the lower images.

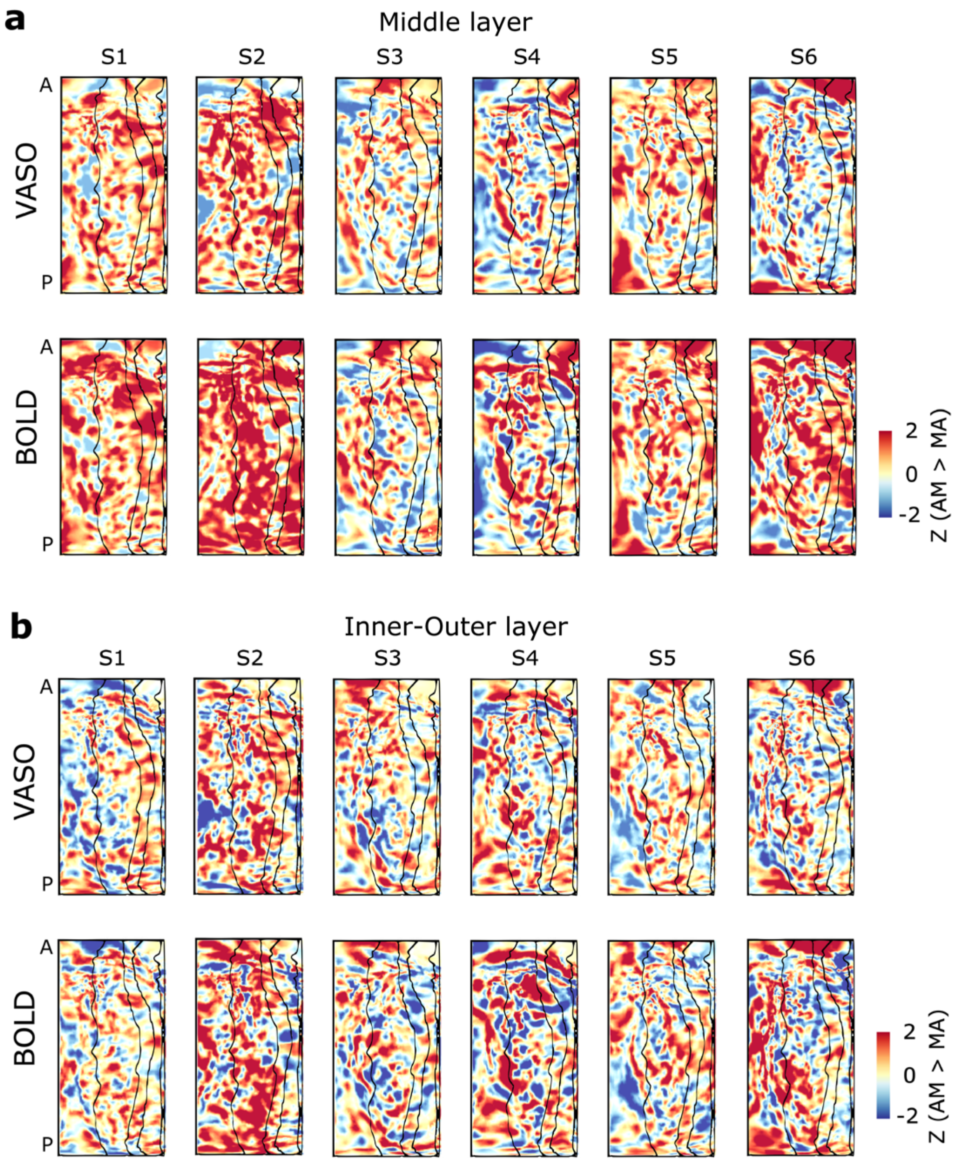

Figure S2 Activation (AM > MA) of individual participants visualized on hippocampal unfolded surfaces without smoothing. (**a**) Activation z-scores projected on the middle of the gray matter layer (equivolume). (**b**) Difference maps between inner and outer layer activation.

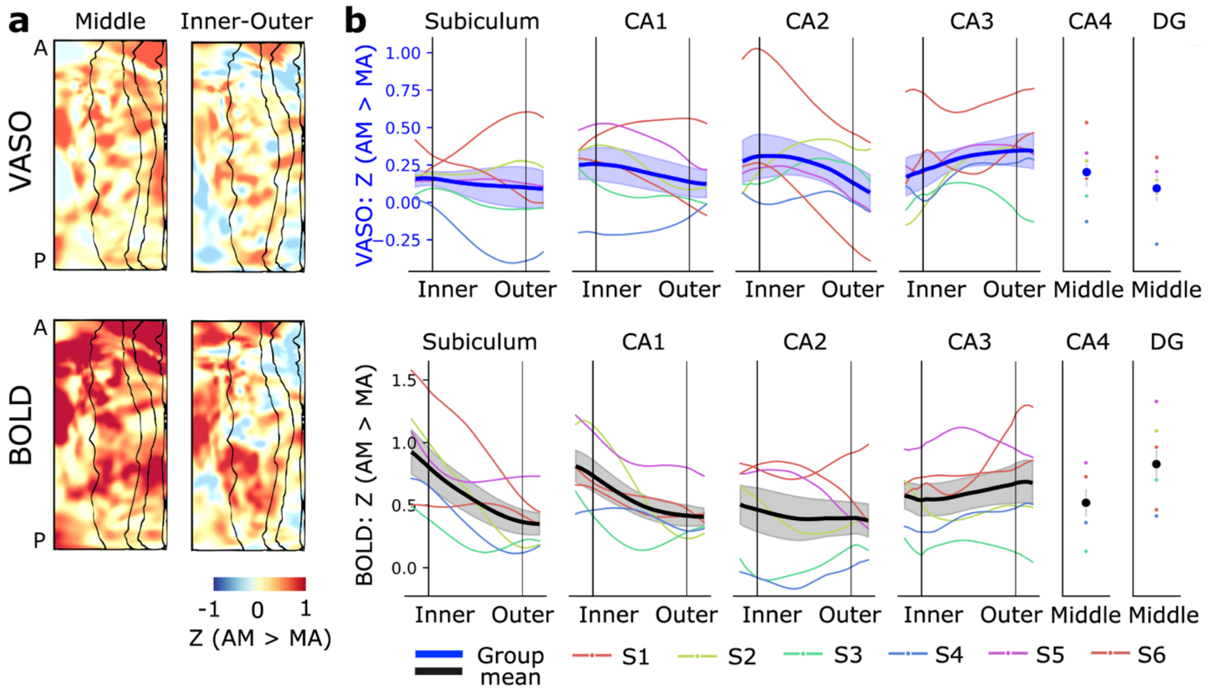

Figure S3 Activation to memory task (AM > MA) with physiological signal regression (aCompCor). (**a**) Group average activation patterns visualized on unfolded surfaces. (**b**) ROI analysis of activation differences to memory stages per hippocampal subfield. Both results replicated main findings without the additional regressors.

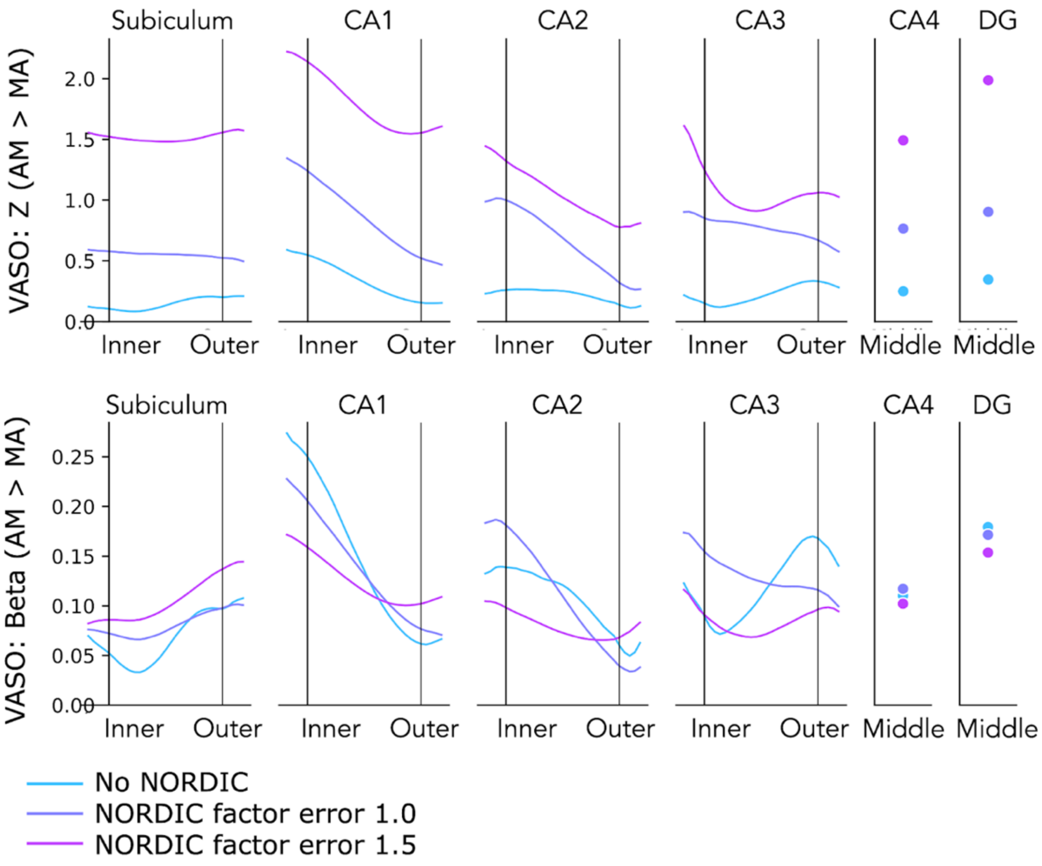

Figure S4 VASO layer profiles (AM > MA) obtained at different factor error levels of NORDIC, in an example participant. NORDIC increased z-score profiles without fundamentally changing the relative profile shape, but some beta estimates (e.g. inner layers of CA1) were reduced.

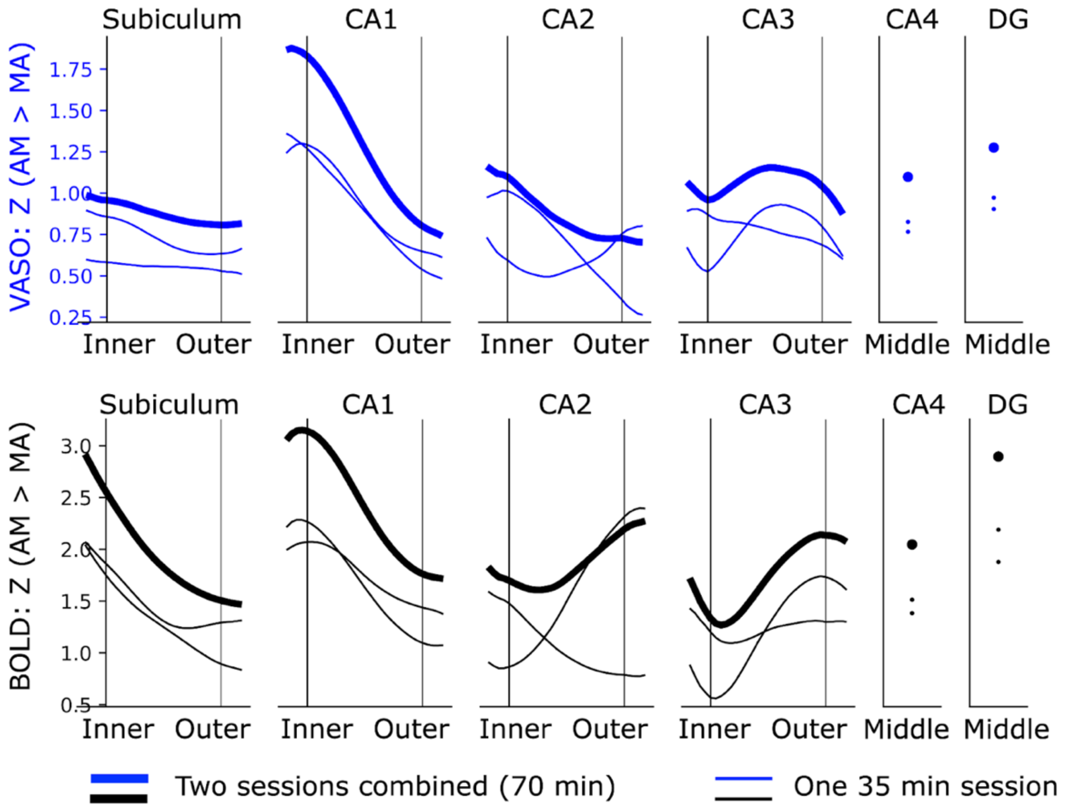

Figure S5 Reliability of the layer profiles in repeat testing in one participant. Layer-specific memory task activation (AM > MA) based on VASO and BOLD from the same acquisition, showing similar profile shapes in subiculum and CA1 during two sessions acquired at different days and their combined analysis.

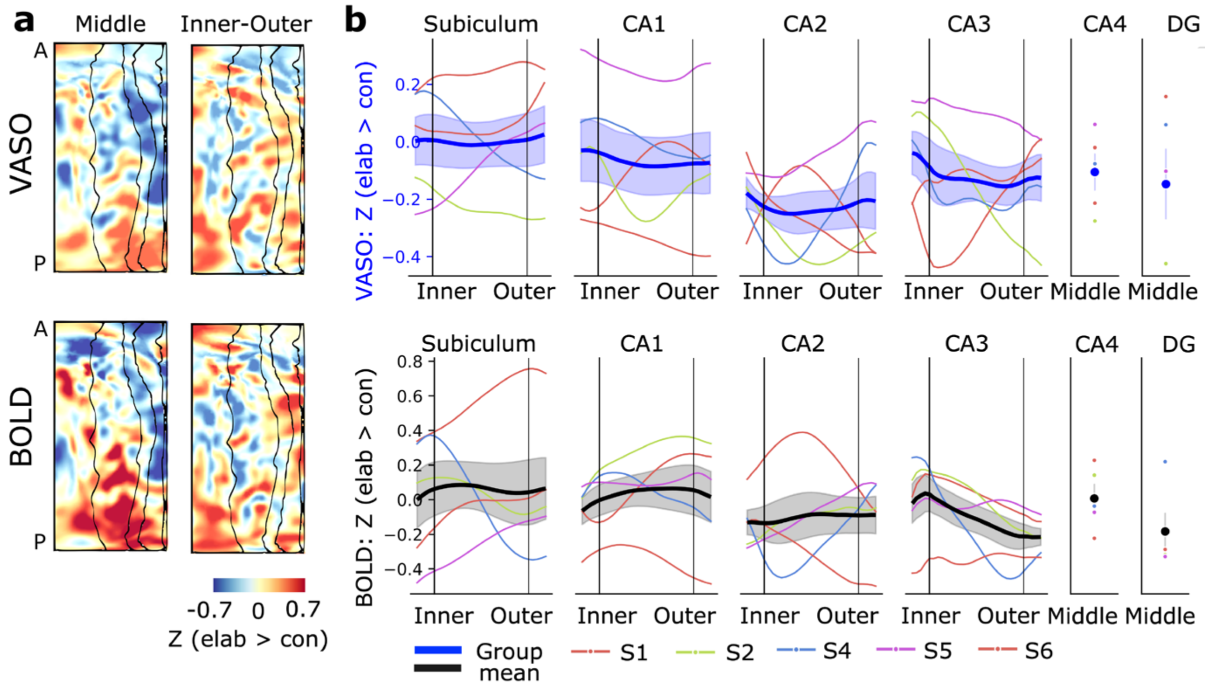

Figure S6 Activation (elaboration > construction) of individual participants visualized on hippocampal unfolded surfaces without smoothing. (**a**) Activation z-scores projected on the middle of the gray matter layer. (**b**) Difference maps between inner and outer layer activation.

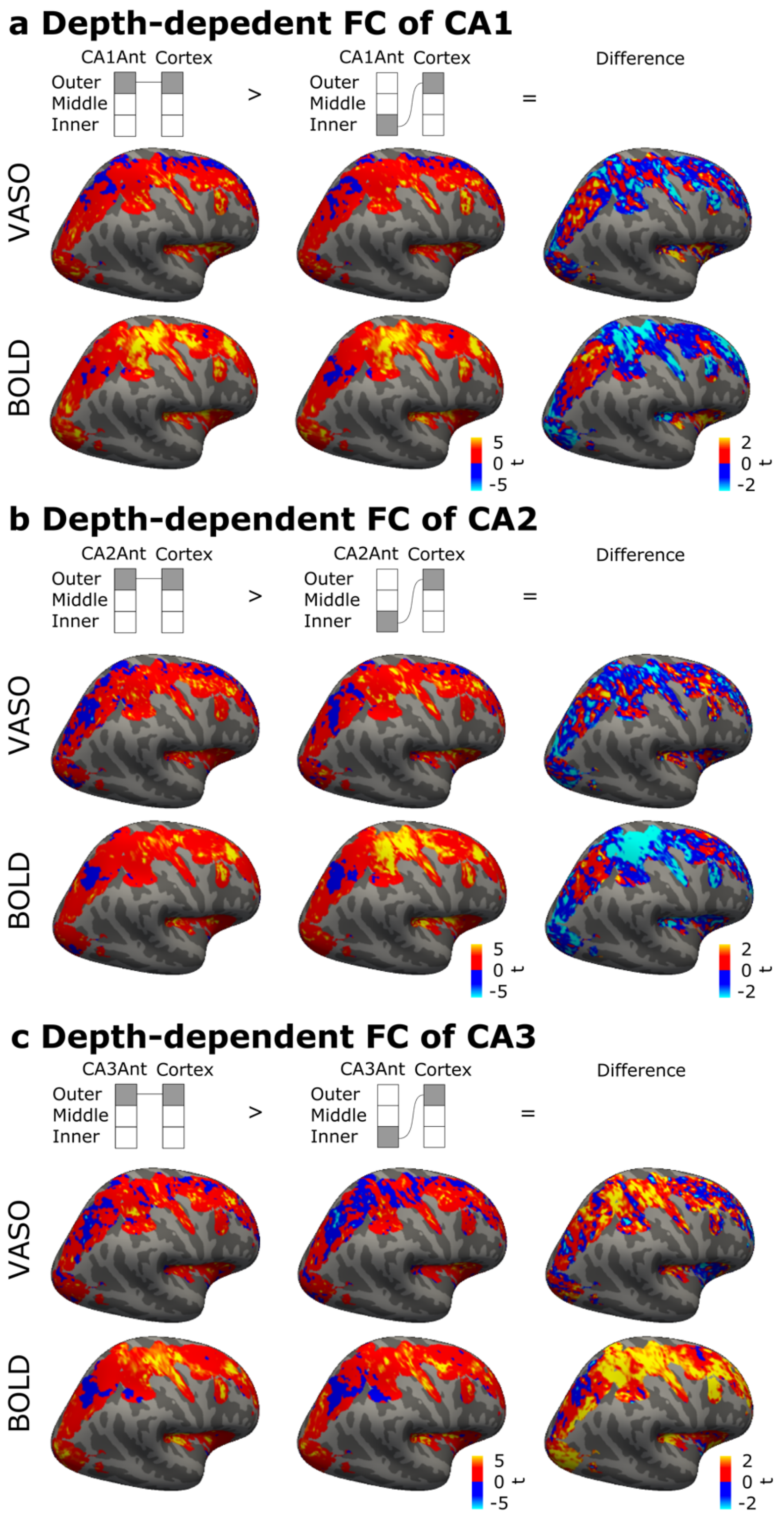

Figure S7 Hippocampal functional connectivity (FC) patterns based on VASO and BOLD. Connectivity to the cortical superficial cortical layer is seeded by inner and outer depths of each hippocampal subfield CA1–3. Statistics are shown only for the right hemisphere vertices imaged in all six participants.
